# Repositioning of new potential schistosomicidal drugs using chemogenomic strategy

**DOI:** 10.1186/1753-6561-8-S4-P55

**Published:** 2014-10-01

**Authors:** Arthur Scalzitti Duarte, José Clecildo Barreto Bezerra, Lourival de Almeida Silva, Bruno Junior Neves, Carolina Andrade, Marina Clare Vinaud, Clélia Christina Mello Silva

**Affiliations:** 1 LAERPH, IPTSP, Federal University of Goiás, Goiânia-GO, Brazil; 2 Instituto Federal Goiano, Campus Ceres, Goiás, Brazil; 3 LabMol, FF, Federal University of Goiás, Goiânia-GO, Brazil; 4 LEE/IOC, Fiocruz, Rio de Janeiro, Brazil

## Background

Schistosomiasis remains a severe problem of public health in developing countries [1]. Several reports show that praziquantel, the drug of choice for treating schistosomiasis, can select *Schistosoma mansoni *strains resistant to the drug. Thus, developing new drugs against this parasitosis is a highly desirable goal [2]. In this context, enzymes involved in energetic metabolism could represent attractive drug targets for novel anti-schistosome chemotherapies [3,4]. We report a chemogenomic strategy for identification of approved drugs that may be able to interfere with energetic metabolism of the *Schistosoma mansoni*.

## Methods

The chemogenomic study was performed on a list containing 734 genes involved in oxidative phosphorylation (n = 45); nitrogen metabolism (n = 642); glycolysis (n = 11); citrate cycle (n = 10); and others (n = 26). Next, it was obtained from the GeneDB *S. mansoni *genome database individual information for genes (amino acid sequence in FASTA format, product name, and biological process). Each of these protein sequences was treated as a potential drug target and used to screen three freely available databases (DrugBank, STITCH 3.1, and TTD) based in the concept of the target sequence similarity. The targets with E-value score ≤ 10^-5 ^and score ≥ 0.7 were considered for further analyses.

## Results and conclusions

We were able to identify several drugs that are expected to interact with 6 targets involved in nitrogen metabolism (carbonic anhydrase II and carbonic anidrase), citrate cycle (succinate dehydrogenase), oxidative phosphorylation (ATP synthase delta chain and NADH-ubiquinone oxidoreductase mitochondrial precursor), and glutamate metabolism (glutaminase). One of these targets was associated with thiabendazole, whose activity has been previously evaluated against *S. mansoni*. [5]. However, 18 drugs were predicted to have activity against other targets and have never been evaluated against schistosoma parasites (e.g., acetazolamide, doxorubicin, morantel tartrate, axantel pamoate, thiabendazole, and menthol). Our next step is to experimentally screen these drugs against *S. mansoni*. Being a cost and time saving route, drug repositioning is expected to accelerate the discovery of new anti-schistosome chemotherapies.

Financial support: FAPEG/Goiás.

## References

[B1] Gryseels B Polman K Clerinx J Kestens L Human schistosomiasis Lancet 2006 368 1106 18 16997665 10.1016/S0140-6736(06)69440-3

[B2] Thétiot-Laurent Sa-L Boissier J Robert A Meunier B Schistosomiasis chemotherapy Angewandte Chemie (International ed in English) 2013 52 7936 56 23813602 10.1002/anie.201208390

[B3] Van Oordt BE Tielens AG Van den Bergh SG The energy metabolism of Schistosoma mansoni during its development in the hamster Parasitology research 1988 75 31 5 3144712 10.1007/BF00931187

[B4] Smith TM Brown JN Tricarboxylic acid cycle enzyme activities in adult Schistosoma mansoni and Schistosoma japonicum Transactions of the Royal Society of Tropical Medicine and Hygiene 1977 71 329 30 595083 10.1016/0035-9203(77)90112-2

[B5] Pancera CF Alves AL Paschoalotti MA Chieffi PP Effect of wide spectrum anti-helminthic drugs upon Schistosoma mansoni experimentally infected mice Revista do Instituto de Medicina Tropical de São Paulo 2013 39 159 63 9460257 10.1590/s0036-46651997000300007

